# Supplementary material for: Geometrically Induced Acceleration for Charging Dynamics of Electrical Double-Layers in a Nanopore with Sloped Walls
Source: J Phys Chem C Nanomater Interfaces. 2026 Mar 4;130(11):4065–79. doi: 10.1021/acs.jpcc.6c00582 (PMC13007040; doi:10.1021/acs.jpcc.6c00582)
Supplement: Supplementary file 1 [file jp6c00582_si_001.pdf]

Geometrically Induced Acceleration for Charging Dynamics of Electrical Double-layers in a Nanopore with Sloped Walls

Bryce Rives,[†] Filipe Henrique,[‡] Paweł J. Żuk,[¶] and Ankur Gupta[*,†]

†*Department of Chemical and Biological Engineering, University of Colorado Boulder, Boulder, CO 80303, USA*

‡*Department of Mechanical and Aerospace Engineering, Princeton University, NJ, USA*

¶*Institute of Physical Chemistry, Polish Academy of Sciences, Warsaw, Poland*

E-mail: ankur.gupta@colorado.edu

Supplementary video 1: We animate the quiver plots of $\boldsymbol{\nabla}\rho(Z,R,\tau)$ and $\boldsymbol{\nabla}\Phi(Z,R,\tau)$ for different κ, representing the diffusive (light pink) and electromigrative (dark blue) fluxes respectively. The entrance-to-end ratio set at 2:1 for the converging pore (a)–(c) and 1:2 for the diverging pore (d)–(f), defined by $\alpha(Z) = 2 - Z$ and $\alpha(Z) = 1 + Z$, respectively. τ denotes the non-dimensional time, Z the non-dimensional axial coordinate, and R the non-dimensional radial coordinate. The initial conditions are $\mu(Z, \tau = 0) = 1$, and $a_{\mathrm{SDL}} = 4$. This video is meant to display the effect of favorable wall direction.

Supplementary figures S1-2: In exploring the electrochemical potential for different values of κ we generate the same plots as before, but with different κ values. First we explore how the electrochemical potential ($\hat{\mu}$) as function of axial position in the overlapping double layer limit or $\kappa = 0.1$. Next, we investigate EDL in the thin limit, or $\kappa = 10$

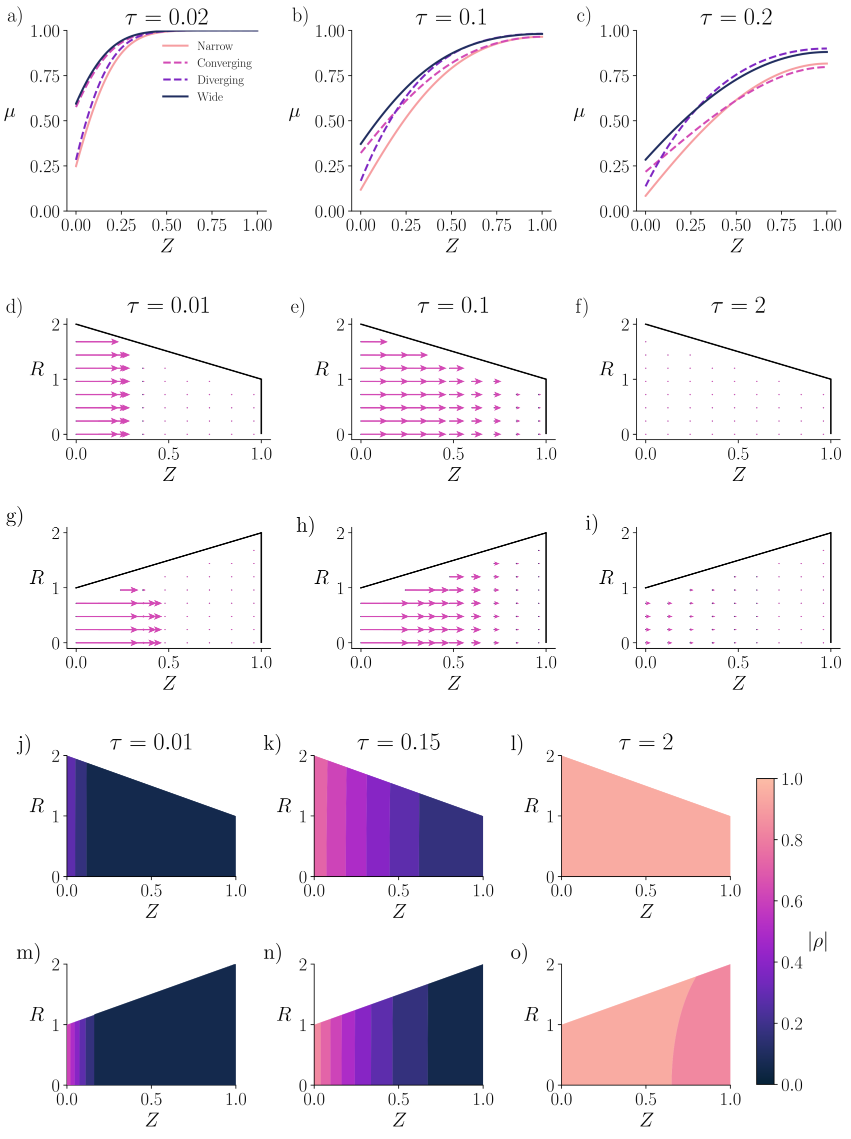

Figure S1: The electrochemical potential ($\hat{\mu}$) plotted at various times for different pore geometries. Electrochemical potential profiles within the pores at early, intermediate, and late times, shown in (a)–(c), respectively. The narrow pore is represented by $\alpha(Z) = 1$, and the wide pore by $\alpha(Z) = 2$. The entrance-to-end ratio for each is 2:1 (converging) or 1:2 (diverging), with corresponding equations $\alpha(Z) = 2 - Z$ and $\alpha(Z) = 1 + Z$, respectively. τ denotes the non-dimensional time, Z the non-dimensional length along the pores, and R the non-dimensional radial length. The initial conditions are $\hat{\mu}(Z, \tau = 0) = 1$, $\kappa = 0.1$, and $a_{\mathrm{SDL}} = 4$. Quiver plot of two driving fluxes: diffusive (light pink) and electromigrative (dark blue). The flux profiles within the pores at early (d)&(g), intermediate (e)&(h), and near equilibrium (f)&(i). Contour plots of charge density (ρ) throughout the pore at various times. (j)–(l) show the converging geometry, and (m)–(o) show the diverging geometry. The charge density contours are shown at early (j)&(m), intermediate (k)&(n), and equilibrium (l)&(o). Lighter regions correspond to higher charge densities, while darker regions represent a more neutral state.

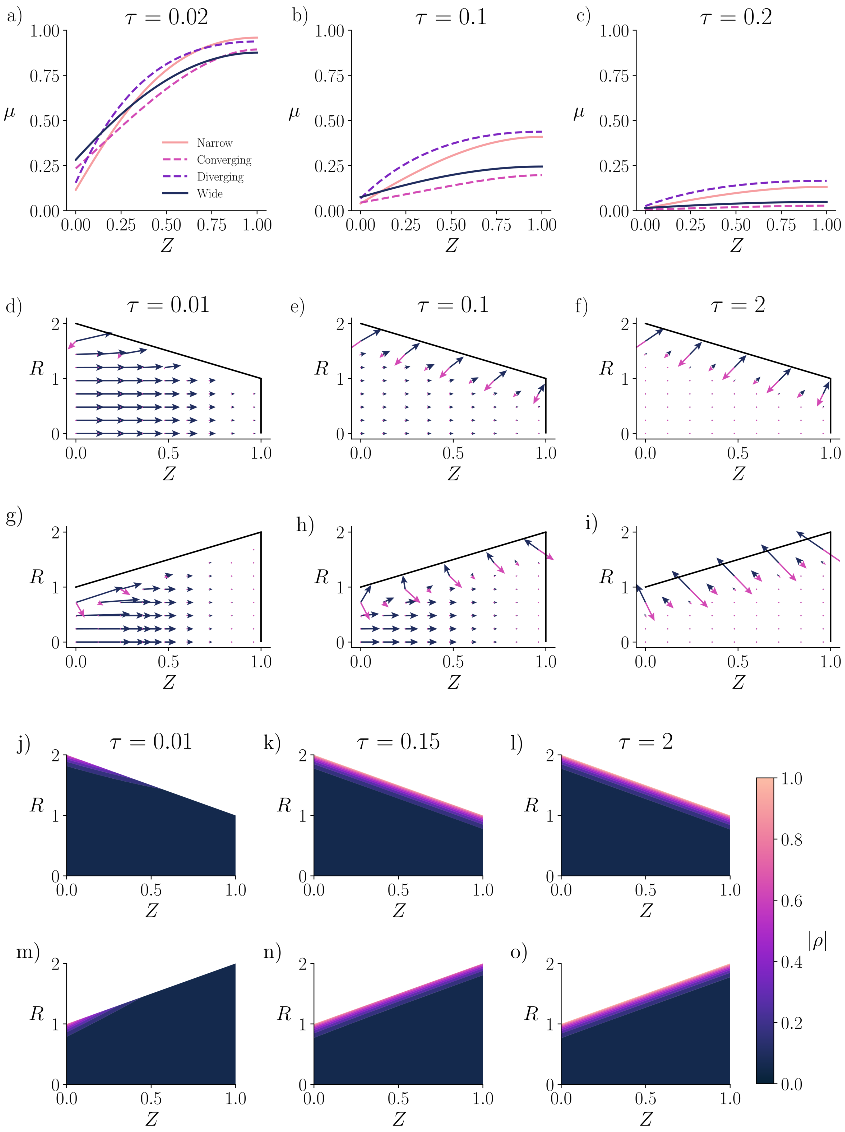

Figure S2: The electrochemical potential ($\hat{\mu}$) plotted at various times for different pore geometries. Electrochemical potential profiles within the pores at early, intermediate, and late times, shown in (a)–(c), respectively. The narrow pore is represented by $\alpha(Z) = 1$, and the wide pore by $\alpha(Z) = 2$. The entrance-to-end ratio for each is 2:1 (converging) or 1:2 (diverging), with corresponding equations $\alpha(Z) = 2 - Z$ and $\alpha(Z) = 1 + Z$, respectively. τ denotes the non-dimensional time, Z the non-dimensional length along the pores, and R the non-dimensional radial length. The initial conditions are $\hat{\mu}(Z, \tau = 0) = 1$, $\kappa = 10$, and $a_{\mathrm{SDL}} = 4$. Quiver plot of two driving fluxes: diffusive (light pink) and electromigrative (dark blue). The flux profiles within the pores at early (d)&(g), intermediate (e)&(h), and near equilibrium (f)&(i). Contour plots of charge density (ρ) throughout the pore at various times. (j)–(l) show the converging geometry, and (m)–(o) show the diverging geometry. The charge density contours are shown at early (j)&(m), intermediate (k)&(n), and equilibrium (l)&(o). Lighter regions correspond to higher charge densities, while darker regions represent a more neutral state.
